# Oxoglutarate dehydrogenase complex controls glutamate-mediated neuronal death

**DOI:** 10.1016/j.redox.2023.102669

**Published:** 2023-03-11

**Authors:** Adelheid Weidinger, Nadja Milivojev, Arthur Hosmann, J. Catharina Duvigneau, Csaba Szabo, Gabor Törö, Laurin Rauter, Annette Vaglio-Garro, Garik V. Mkrtchyan, Lidia Trofimova, Rinat R. Sharipov, Alexander M. Surin, Irina A. Krasilnikova, Vsevolod G. Pinelis, Laszlo Tretter, Rudolf Moldzio, Hülya Bayır, Valerian E. Kagan, Victoria I. Bunik, Andrey V. Kozlov

**Affiliations:** a Ludwig Boltzmann Institute for Traumatology, The Research Center in Cooperation with AUVA, Vienna, Austria; b Austrian Cluster for Tissue Regeneration, Vienna, Austria; c Department of Neurosurgery, Medical University of Vienna, Vienna, Austria; d Institute for Medical Biochemistry, University of Veterinary Medicine Vienna, Vienna, Austria; e University of Fribourg, Section of Science and Medicine, Department of Oncology, Microbiology and Immunology, Section of Pharmacology, Fribourg, Switzerland; f Department of Anesthesiology, University of Texas Medical Branch, Galveston, TX, USA; g A. N. Belozersky Institute of Physicochemical Biology, Lomonosov Moscow State University, 119234, Moscow, Russia; h Faculty of Bioengineering and Bioinformatics, Lomonosov Moscow State University, Moscow, Russia; i Department of Biochemistry, Sechenov University, Moscow, Russia; j Biological Faculty, Department of Biophysics, Lomonosov Moscow State University, Moscow, Russia; k Institute of General Pathology and Pathophysiology, Laboratory of Fundamental and Applied Problems of Pain, Moscow, Russia; l National Medical Research Center of Children's Health, Russian Ministry of Health, Laboratory of Neurobiology and Brain Development, Moscow, Russia; m Department of Biochemistry, Semmelweis University, Budapest, Hungary; n Departments of Environmental and Occupational Health, Pharmacology and Chemical Biology, Chemistry and Center for Free Radical and Antioxidant Health University of Pittsburgh, Pittsburgh, PA, USA; o Department of Critical Care Medicine, Safar Center for Resuscitation Research, Children's Neuroscience Institute, Children's Hospital of Pittsburgh, University of Pittsburgh, Pittsburgh, PA, USA

## Abstract

Brain injury is accompanied by neuroinflammation, accumulation of extracellular glutamate and mitochondrial dysfunction, all of which cause neuronal death. The aim of this study was to investigate the impact of these mechanisms on neuronal death.

Patients from the neurosurgical intensive care unit suffering aneurysmal subarachnoid hemorrhage (SAH) were recruited retrospectively from a respective database. *In vitro* experiments were performed in rat cortex homogenate, primary dissociated neuronal cultures, B35 and NG108-15 cell lines. We employed methods including high resolution respirometry, electron spin resonance, fluorescent microscopy, kinetic determination of enzymatic activities and immunocytochemistry.

We found that elevated levels of extracellular glutamate and nitric oxide (NO) metabolites correlated with poor clinical outcome in patients with SAH. In experiments using neuronal cultures we showed that the 2-oxoglutarate dehydrogenase complex (OGDHC), a key enzyme of the glutamate-dependent segment of the tricarboxylic acid (TCA) cycle, is more susceptible to the inhibition by NO than mitochondrial respiration. Inhibition of OGDHC by NO or by succinyl phosphonate (SP), a highly specific OGDHC inhibitor, caused accumulation of extracellular glutamate and neuronal death. Extracellular nitrite did not substantially contribute to this NO action. Reactivation of OGDHC by its cofactor thiamine (TH) reduced extracellular glutamate levels, Ca^2+^ influx into neurons and cell death rate. Salutary effect of TH against glutamate toxicity was confirmed in three different cell lines.

Our data suggest that the loss of control over extracellular glutamate, as described here, rather than commonly assumed impaired energy metabolism, is the critical pathological manifestation of insufficient OGDHC activity, leading to neuronal death.

## Highlights

• OGDHC is more susceptible to the inhibition by NO than mitochondrial respiration.
• OGDHC regulates extracellular glutamate levels and their toxic effects.
• Impaired oxidative phosphorylation is secondary to glutamate toxicity.
• TH can reactivate OGDHC and rescue neurons from glutamate mediated death.

## Graphical abstract

**Figure:**
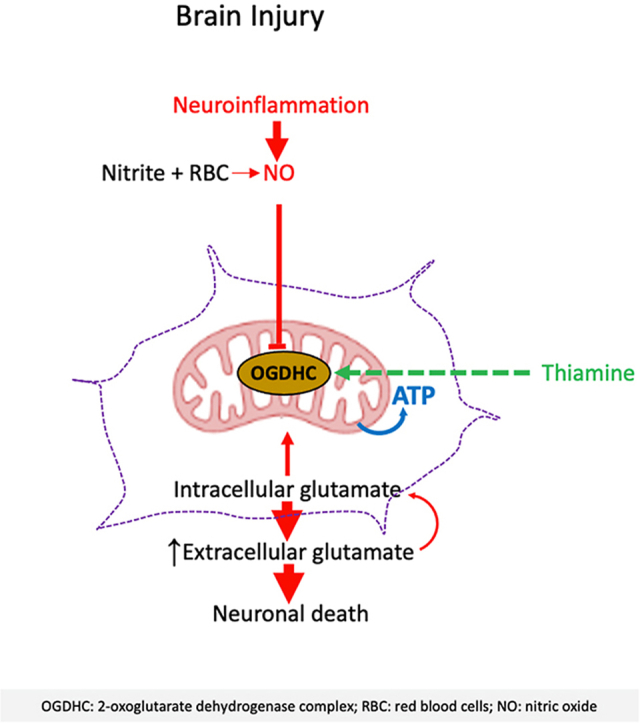

## 1 Introduction

Mitochondrial dysfunction, neuroinflammation and glutamate toxicity are associated with brain injury and neurodegenerative diseases. Mitochondrial dysfunction has been repeatedly reported for most neurodegenerative diseases [1,2] and acute brain injury (Yang, 2016). Moreover, mitochondrial dysfunction has been associated with increased nitric oxide (NO) generation from neuroinflammatory sources [[3], [4], [5]]. The major sources of NO in the brain are immune cells activated by neuroinflammation accompanying both acute brain injury and neurodegenerative diseases. NO has multiple targets within mitochondria, including several enzymes of the tricarboxylic acid (TCA) cycle [6], complexes of electron transport chain (ETC) [7], and mitochondrial permeability transition pore (mPTP) [8]. However, experiments in which these effects were found employed very high concentrations of NO, which are biologically irrelevant. Notably, direct measurements of NO in the rat brain tissue after acute brain injury showed that its concentration was as low as 4 μmol/kg (4 nmol/g) [9]. In clinical settings, only indirect detection of NO is available. The total amount of NO metabolites (nitrite plus nitrate) in patients with acute brain injury (first 7 days) can be determined by means of microdialysis from affected parts of the brain. NO has been found at levels of approximately 100 μM [10] declining below 40 μM after the completion of the acute phase of brain injury. These concentrations are still substantially higher than corresponding concentrations of NO metabolites in the blood, which are approximately 15 μM [11].

It has been shown that elevated NO levels correlate with increased extracellular glutamate levels [10]. Thisis associated with the activation of glutamate receptors [12], and causes excitotoxicity and neuronal death [13]. Physiological and excitotoxic effects of glutamate are mediated by two different groups of glutamate receptors, synaptic and extrasynaptic, respectively [14,15]. The cytotoxic effect of glutamate can be counteracted by reducing its extracellular concentration via uptake into astrocytes and neurons. The major glutamate transporter, glutamate transporter-1 (GLT-1), is highly expressed in astrocytes but also in neurons. The function of neuronal GLT-1 is incompletely understood [16,17], but it is known that in neurons GLT-1-mediated glutamate uptake provides glutamate predominantly for energy metabolism [17], while neurotransmission is supported by glutamine/glutamate cycle. However, it has been shown that the rate of glutamate absorption in neurons also can regulate neurotransmission [[18], [19], [20]]. The rate of glutamate absorption is regulated by GLT-1 diffusion changing its local densities [18]. In astrocytes, GLT-1 mediated glutamate uptake regulates extracellular glutamate persistence and duration of NMDA receptor activation in neurons [18]. Not only glutamate uptake, but also spontaneous glutamate release from the synapses through the plasma membrane can aggravate excitotoxic effects of glutamate [21]. More recently, glutamate-mediated cell death was shown to be also associated with an inhibition of cystine/glutamate antiporter and induction of ferroptosis [22].

In our previous studies, we have shown an increase in NO metabolites in preclinical model of acute traumatic brain injury [9] and in patients during the acute phase of subarachnoid hemorrhage (SAH). Since glutamate metabolism is linked to mitochondrial function and mitochondrial function is modulated by NO, we hypothesize that NO is capable to inhibit 2-oxoglutarate dehydrogenase complex (OGDHC) and thereby limit glutamate consumption, since in the TCA cycle, OGDHC facilitates the turnover of 2-oxoglutarate, derived from glutamate by glutamate dehydrogenase (GDH). Consequently, glutamate accumulates in the extracellular space and causes neuronal cell death. In order to elucidate this mechanism, we investigated the association of NO with extracellular glutamate in animal models and patients suffering from SAH. Using cell culture models, we studied the cellular uptake of glutamate into mitochondria and the activity of OGDHC-mediated glutamate consumption. Previously, we have demonstrated that the mitochondrial OGDHC is crucial in glutamate excitotoxicity [23,24]. Our previous studies have also identified succinyl phosphonate (SP) as a highly selective inhibitor of the isoenzymes of the first component of the complex [25,26]. The involvement of OGDHC was dissected in neuronal culture using its coenzyme thiamine (TH) and inhibitor SP. In mitochondria TH is not only the coenzyme of OGDHC, but also of pyruvate dehydrogenase (PDH). In contrast, SP selectively inhibits OGDHC [27]. Preincubation of cerebellar granule neurons with this analog of 2-oxoglutarate was shown to protect the cells from glutamate-induced Ca^2+^ dysregulation and irreversible mitochondrial depolarization, followed simultaneously by fluorescence of Fura-2FF and rhodamine 123, respectively [23,24]. In the current work, we demonstrate that TH, the precursor of the OGDHC cofactor thiamine pyrophosphate (TPP), has an ability to restore NO-disturbed OGDHC function, normalize glutamate levels and prevent cell death, while SP caused accumulation of extracellular glutamate and neuronal death. We thus provide molecular mechanisms for the positive action of TH that were earlier observed in the rat models of neurotrauma [[28], [29], [30]]. We propose that TH and/or its currently available pharmacological forms may be utilized in an experimental therapeutic strategy to ameliorate glutamate excitotoxicity.

## 2 Results

**Fig. 1:**
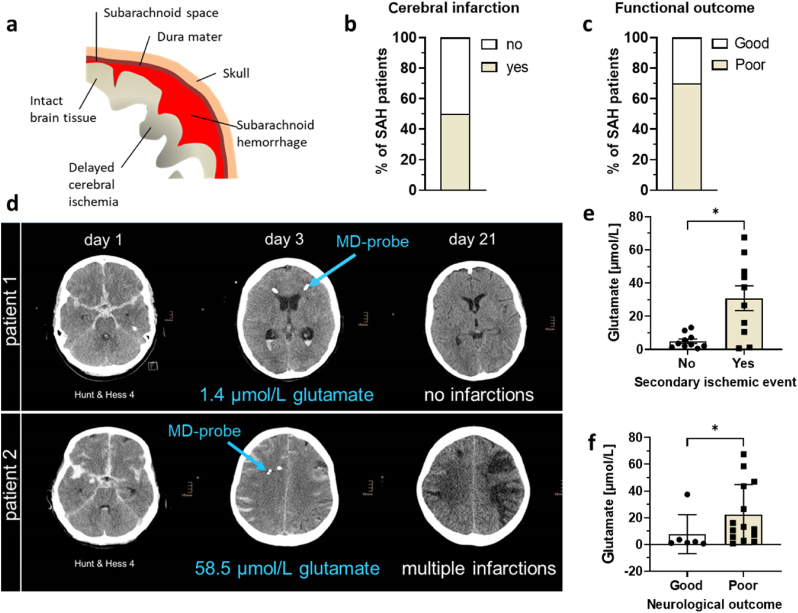
Correlation between intracranial glutamate and clinical outcome in patients suffering severe aneurysmal SAH. (**a**) Schematic presentation of SAH; (**b**) percentage of SAH patients with manifestation of cerebral infarction and (**c**) poor functional outcome 3 months after the bleeding used in this study. Images **d** represent two patients suffering severe aneurysmal SAH, both Hunt&Hess grade IV, revealed distinct clinical course. In case patient 1 (**d**), cerebral microdialysis (MD) showed glutamate levels of 1.4 μmol/L following the period of early brain injury (day 3 after SAH). In this patient, no secondary ischemic events on computed tomography (CT) scans were observed and the patient showed good recovery with a modified Rankin Scale (mRS) of 1 at 3 months. In case patient 2 (**d**) cerebral glutamate was as high as 58.5 μmol/L on day 3. In this patient, multiple cerebral infarctions occurred within the following days, resulting in poor functional outcome (mRS 5) 3 months following SAH. (**e**) Correlation between cerebral glutamate levels on day 3 following SAH and either secondary ischemic events or (**f**) neurological outcome. *p < 0.05. Statistical significance was evaluated by t – test.

**Correlation between brain NO, glutamate levels and clinical outcome in SAH patients.** In our previous studies, we have shown an increase in NO metabolites in preclinical model of acute traumatic brain injury [9] and in patients during the acute phase of SAH [10]. In the current study we explore the pathophysiological consequences of these observations. The impact of aneurysmal SAH causes early brain injury, which induces secondary pathophysiological processes, leading to microcirculatory hypoperfusion and delayed cerebral ischemia (Fig. 1a), resulting in poor functional outcome. We have measured the concentration of glutamate in microdialysates obtained from brain tissue at risk for secondary ischemic events in patients suffering from SAH in the early phase of this disease. Measurement of brain glutamate levels during early brain injury (day 3 following SAH) revealed a strong relationship of elevated glutamate with delayed cerebral ischemia and poor clinical outcome (Fig. 1). Our study utilized twenty patients suffering severe aneurysmal SAH and assessed acute events (infarction) and neurological outcomes. Cerebral infarction within the first 21 days after admission was observed in 10 patients (50%, Fig. 1b). Median modified Rankin Scale (mRS) at 3 months was 4 (interquartile range (IQR): 2–5). Functional outcome was poor in 70% of patients (Fig. 1c). Mean cerebral glutamate levels on day 3 following SAH were 18.0 ± 21.2 μmol/L. Patients developing delayed cerebral ischemia on days 4–21 had significantly higher extracellular cerebral glutamate levels (30.9 ± 23.6 μmol/L) on day 3 than patients without cerebral infarctions (5.0 ± 4.3 μmol/L, p = 0.02; Fig. 1e). Likewise, patients with poor functional outcome had significantly higher cerebral glutamate levels (22.4 ± 22.5 μmol/L) on day 3 than patients with good recovery (7.7 ± 14.6 μmol/L, p = 0.03; Fig. 1f).

In Fig. 1d we present two patient cases with good and poor functional outcome. One patient had a good functional outcome without signs of infarction and without significant disability (Fig. 1d, upper panel), while the other patient developed multiple infarctions by three weeks after admission and severe disability (Fig. 1d, bottom panel). The glutamate concentration by third day after SAH in the patient 1 was 1.4 μmol/L, while in the second patients it was as high as 58.5 μmol/L.

Our previous work [10] has demonstrated a positive correlation between glutamate and NO metabolites in the brain. Combined with the current data this lead us to an assumption that elevated NO levels impair glutamate metabolism and that elevated glutamate levels were associated with the negative clinical outcome.

Therefore, we have designed mechanistic studies to determine the potential mechanism(s) underlying this impairment.

**Fig. 2:**
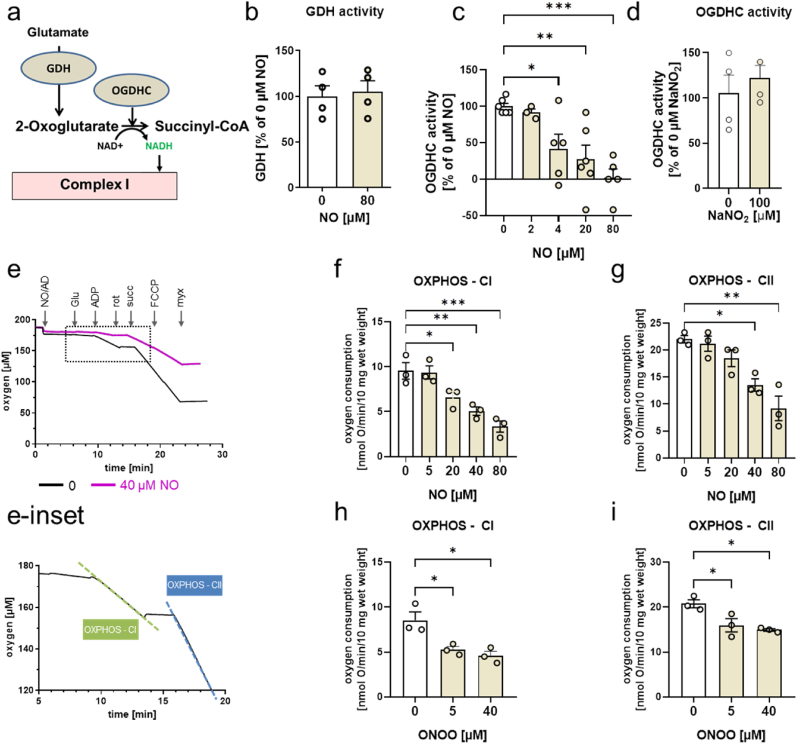
**Effects of NO on the TCA cycle enzymes and mitochondrial respiration in rat cortex homogenates.** (**a**) Glutamate-dependent segment of the TCA cycle examined in these experiments; (**b**) and (**c**) effect of NO on GDH and OGDHC activities, respectively; (**d**) effect of nitrite on OGDHC activity; (**e**) and (**e-inset**) experimental procedure for examination of mitochondrial function; (**f**), (**g**) effect of NO on the mitochondrial oxidative phosphorylation (OXPHOS) via complex I (**f**) and complex II (**g**); (**h**), (**i**) - effect of peroxynitrite (ONOO^−^) on OXPHOS via complex I (**h**) and complex II (**i**). Enzymatic activities were determined by generation of NADH in the presence of corresponding substrates. OXPHOS was determined by oxygen uptake upon addition of ADP to mitochondria respiring with 10 mM glutamate (complex I substrate) or 10 mM succinate (complex II substrate). St2 – state 2 respiration of glutamate; St-3-CI and St-3-CII, state 3 respirations determined for complex I (OXPHOS CI) and complex II (OXPHOS CII), respectively. See also methods section for details. The data were analyzed by either one-way ANOVA with Dunnett's multiple comparisons test or two-tailed *t*-test (for two groups). Data are presented as mean ± SEM, n ≥ 3, * - p < 0.05; /**p < 0.01/*** - p < 0.001 *vs*. 0 μM).

**NO targets in cortex responsible for impaired glutamate metabolism.** Since glutamate metabolism is linked to mitochondrial function, which, in turn, is modulated by NO, we examined effects of NO and its derivatives on the enzymes of the glutamate-dependent segment of the TCA cycle (OGDHC) affiliated with GDH and oxidative phosphorylation (OxPhos) (Fig. 2a). We found that the activity of GDH was unaffected (Fig. 2b), while OGDHC activity was markedly inhibited by 4 μM NO (Fig. 2c). In contrast, 100 μM of NO_2_^−^ did not affect OGDHC activity (Fig. 2d). We did not examine NO_3_^−^, because it does not have any biological effect in mammalian cells [11]. OxPhos, determined by standard polarography methods (Fig. 2e and 2e-inset), was also impaired by NO, but by substantially higher NO concentrations than OGDHC both with glutamate (Fig. 2f) and with succinate, a substrate for complex II (Fig. 2g). In contrast to NO, 5 μM peroxynitrite (ONOO^−^), a product of reaction between NO and O_2_^−^, inhibited both complex I (glutamate oxidation by native mitochondria, Fig. 2h) and complex II (succinate oxidation by native mitochondria, Fig. 2i). Thus, the NO at low concentrations can only inhibit OGDHC, as described above. This blocks the consumption of glutamate by the native mitochondria facilitating its accumulation in cytoplasm, followed by the spontaneous glutamate release into the extracellular pool, inducing cell death.

**Fig. 3:**
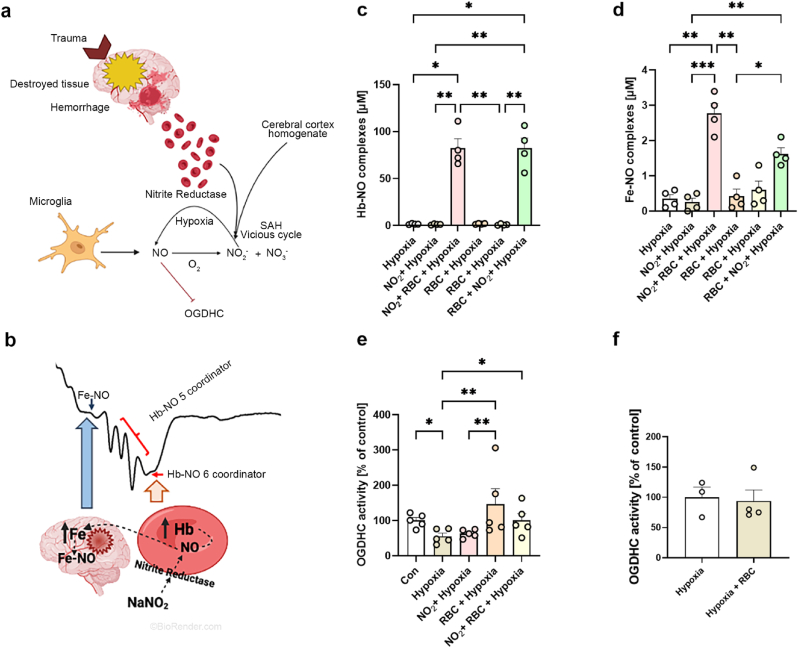
**The impact of RBC on the OGDHC activity.** (**a**) Pathways of NO generation in brain tissue in the presence of RBC. (**b**) Determination of NO bound to hemoglobin (Hb-NO) and released in cortex homogenate determined by formation of nitrosyl complexes of hemoglobin and iron ions (Fe–NO). The arrows indicate the peaks corresponding to Fe–NO and Hb-NO, respectively. Concentrations of (**c**) Hb-NO (**d**) and Fe–NO in the brain homogenate (BH), effects of hypoxia and the presence of RBC and nitrite. (**e**) The OGDHC activity upon hypoxia, effect of RBC and nitrite. (**f**) RBC added after hypoxia do not influence the activity of OGDHC. The data are presented as mean ± SEM, n = 3–5. The data were analyzed by Kruskal-Wallis followed by Dunn's test, *p < 0.05, **p < 0.01, ***p < 0.001. Schematic representation created with biorender.com.

In SAH patients, we observed high levels of NO metabolites [10]. The major sources of NO in tissues are a family of NO synthases and nitrite as the major NO metabolite, which however can be reduced back to NO by nitrite reductases (Fig. 3a). Since both high nitrite levels and hemorrhage occur in SAH patients, one can expect that the release of additional NO from nitrite catalyzed by nitrite reductase activity of red blood cells (RBC) can further aggravate OGDHC dysfunction. To test this assumption we examined NO release and OGDHC activity in rat cortex homogenate in the presence of RBC and nitrite at concentrations observed in patients. Electron paramagnetic resonance (EPR) spectroscopy experiments (Fig. 3b) demonstrate that NO is released from nitrite but the majority of NO remains within RBC in a form NO-Hb (Fig. 3c). Only a small portion of NO is released forming Fe–NO complexes (Fig. 3d). The amount of released NO was much lower than 4 μM and we did not observe significant effects on OGDHC activity (Fig. 3e), which is in a good agreement with the previous experiments. Of note, in this experiment we observed an inhibition of OGDHC induced by hypoxia which was reversed by RBC (Fig. 3e). This effect did not appear if RBC were added after hypoxia (Fig. 3f).

**Fig. 4:**
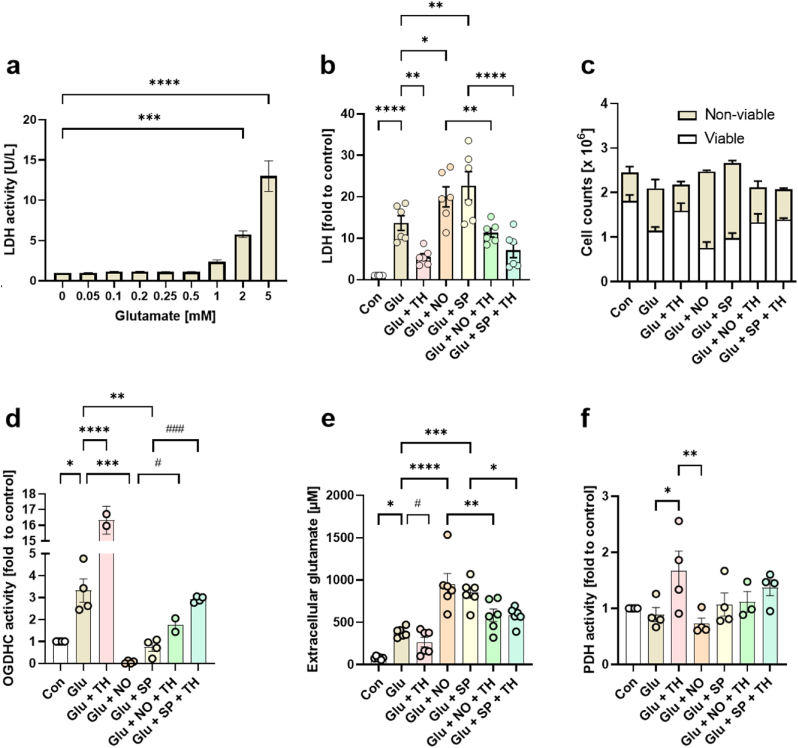
Influence of glutamate accumulation on OGDHC activity and consequent toxicity in NG108-15 cells. (**a**) Effect of increasing glutamate concentrations on the release of LDH. (**b**) Effect of glutamate, NO, SP and TH on LDH release, (**c**) non-viable cell counts, (**d**) OGDHC activity, (**e**) extracellular glutamate concentrations and (**f**) PDH activity. Cells were treated with 5 mM glutamate, 1 mM TH, 0.5 mM NO-donor DETA-NONOate and 0.2 mM SP over 48 h. Cell pellets were collected for determination of viability, OGDHC and PDH activity; culture supernatants were collected for detection of extracellular glutamate concentration and LDH release. The data are presented as mean ± SEM. The data were analyzed by either one-way ANOVA with Tukey's multiple comparisons test, *p < 0.05, **p < 0.01, ***p < 0.001, ****p < 0.0001 or by paired two tailed *t*-test, #p < 0.05, ###p < 0.001.

**Mechanism of NO toxicity mediated by impaired OGDHC activity.** To further investigate the mechanism and interactions between NO, OGDHC and glutamate toxicity we used NG108-15 cells (mouse neuroblastoma cell line expressing NMDA receptors) [31]. Although this cell line contains less receptors compared to primary neurons, the cells can be maintained without astrocytes and respond to the cytotoxic effect of glutamate administration. First, we determined the threshold and time course of glutamate-induced cytotoxic effects in this cell line using the release of lactate dehydrogenase (LDH) as a marker for cell damage (Fig. 4a). For subsequent experiments we have selected 5 mM glutamate, which resulted in the death of nearly 50% of all neurons within 48 h. We used TH, NO, and SP to modulate enzymatic activity of OGDHC. We observed that the release of LDH induced by glutamate was reduced by TH (Fig. 4b) and elevated by SP and NO (Fig. 4b). Similar results were obtained if the count of non-viable cells was considered instead of LDH (Fig. 4c).

OGDHC activity was increased upon treatment with glutamate; this effect was particularly strong when glutamate was combined with TH (Fig. 4d). In contrast, adding NO and SP to glutamate inhibited the activity of OGDHC. Further supplementation of the cells with TH partially restored OGDHC activity in presence of SP and NO with glutamate (Fig. 4d). The lowest levels of extracellular glutamate, close to control values, were observed when glutamate treatment was combined with TH, while NO and SP drastically increased the levels of resting extracellular glutamate. Supplementation with TH lowered extracellular glutamate levels, which were raised by NO and SP (Fig. 4e). Generally, the release of both extracellular glutamate and LDH displayed an increase when OGDHC activity was inhibited, whereas the opposite was shown when the enzyme activity was enhanced. These data imply that TH may be capable of restoring impaired OGDHC activity and counteracting glutamate release and cell death.

Since TH is a cofactor not only for OGDHC in the TCA cycle, but also for PDH, we next tested if changes in PDH activity were associated with the toxic effects of glutamate in neurons. We observed that only TH supplementation increased PDH activity, while no other treatment (NO, SP) affected it (Fig. 4f). These findings suggest that the beneficial effects of TH are due to the activation of OGDHC, rather than the activation of PDH.

**Fig. 5:**
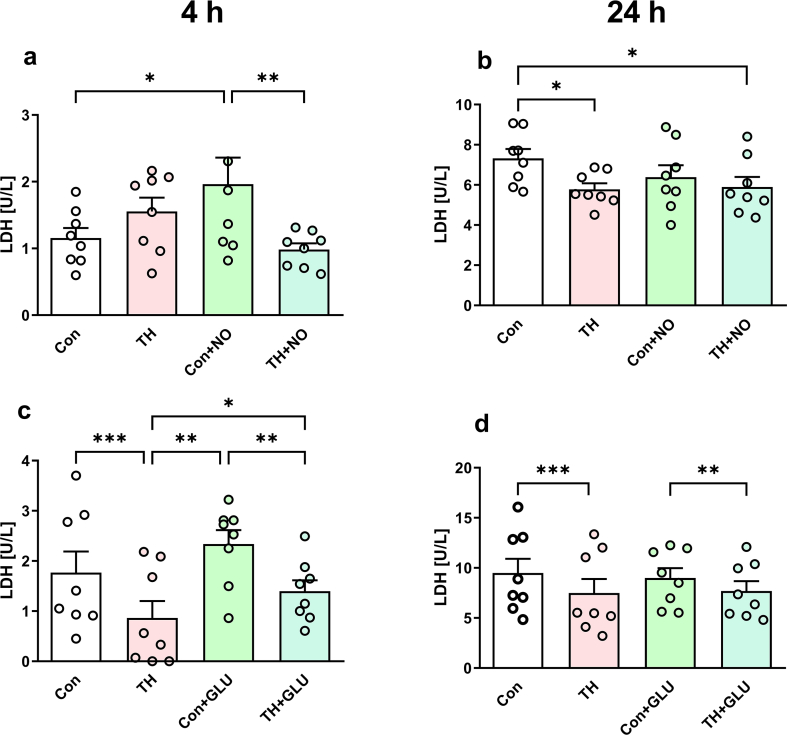
Effect of the OGDHC enzyme precursor TH on LDH released from neurons (B35 cell line) treated with NO or glutamate. (**a**) Effect of TH on the LDH release in control cells and cells treated with NO by 4 h after treatment with NO; (**b**) effect of TH on the LDH release in control cells and cells treated with NO by 24 h after treatment with NO; (**c**) effect of TH on the LDH release in control cells and cells treated with glutamate by 4 h after treatment with glutamate; (**d**) effect of TH on the LDH release in control cells and cells treated with glutamate by 24 h after treatment with glutamate. One-way ANOVA with Fisher's LSD test. The data are presented as mean ± SEM; n = at least 6, *p < 0.05, **p < 0.01, ***p < 0.001.

**Fig. 6:**
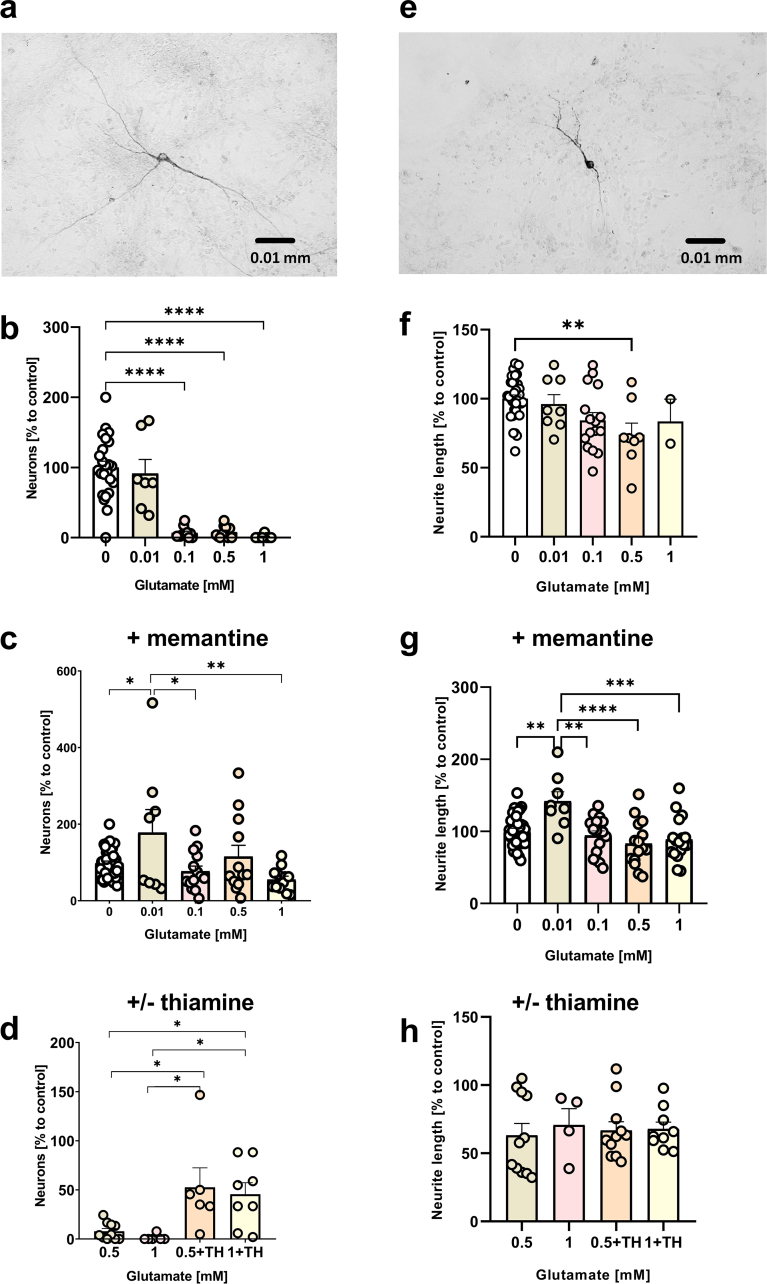
Number of dopaminergic (THir positive) neurons and neurite outgrowth in murine mesencephalic cultures. (**a**) Typical morphology of intact neurons. (**b**) Glutamate (15 min treatment) substantially reduced the number of neurons. (**c**) This effect was attenuated by addition of memantine (60 μM, 75 min in general, added 60 min before glutamate), an NMDA receptor antagonist. (**d**) Addition of thiamine (1 mM, 75 min in general, added 60 min before glutamate) remarkably increased the survival rate of primary neuronal cells. (**e**) The length of neurites had a trend to become shorter in glutamate-treated THir neurons (0.5 mM). (**f**) This shortening was significant at 0.5 mM of glutamate. (**g**) In the presence of memantine the length of neurons was increased at 0.01 mM of glutamate; no other changes or trends were observed upon this treatment. (**h**) Addition of thiamine did not affect the length of neurons. The data were tested by ROUT test (Q = 5%) for outliers and analyzed by one-way ANOVA with Holm-Sidak multiple comparisons test. Data are presented as mean ± SEM; *p < 0.05, **p < 0.01, ***p < 0.001, ****p < 0.0001.

**Beneficial effects of TH in other neuronal cultures.** Beneficial effects of TH were also confirmed using B35 cells, a rat neuroblastoma cell line, which displays a different sensitivity to glutamate. Therefore, treatments were adjusted for that type of cells. Our data show that NO or glutamate both increase the release of LDH while TH reverts these effects. This protective effect was observed after 4 h (Fig. 5 a,c) and 24 h (Fig. 5 b,d) after treatment with either NO (Fig. 5 a,b) or glutamate (Fig. 5 c,d). Salutary effects of TH on glutamate toxicity were also confirmed in primary rat murine mesencephalic cells. The majority of cells were killed already after 15 min incubation with 0.5 and 1 mM of glutamate (Fig. 6b). This effect was substantially reduced by the addition of memantine, an NMDA receptor antagonist (Fig. 6c,g) suggesting that the death pathway is mediated by this receptor. Furthermore, the typical morphology of intact neurons (Fig. 6a) was altered upon treatment with glutamate (Fig. 6e) reducing the length of neurites and decreasing the number of tyrosine hydroxylase immunoreactive cells (Fig. 6b,f). TH increased the number of surviving neurons (tyrosine hydroxylase immunoreactive cells) from 22.8 to 64.7% in cultures treated with 0.5 mM glutamate, and from 18.6% to 45.6% in cultures treated with 1 mM glutamate. However, TH did not normalize the length of neurons (Fig. 6 d,h).

**Fig. 7:**
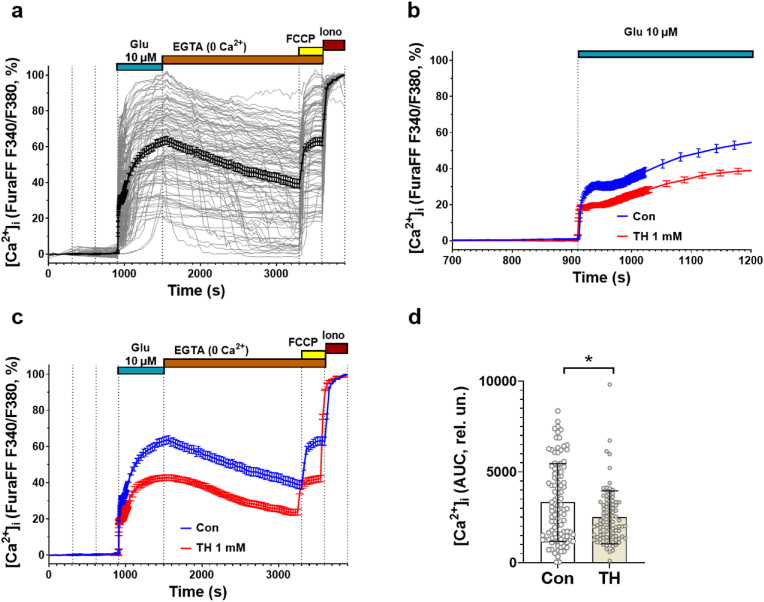
The effect of thiamine (TH) on the glutamate induced changes in intracellular free Ca^2+^ concentration ([Ca^2+^]_i_). (**a**) Changes in [Ca^2+^]_i_ in individual neurons (thin grey curves) and average traces (bold lines) in control culture (glutamate without TH) (n = 101 cells; error bars correspond to SEM). (**b**) X-scale expansion of [Ca^2+^]_i_ changes during the first 2 min of glutamate administration ([Ca^2+^]_i_ signals were monitored at 0.8 Hz frequency; the rest of experiment the recordings were carried out every 30 s). (**c**) Average changes of [Ca^2+^]_i_ (Mean ± SEM) of neurons in response to glutamate (glutamate, 10 μM) application in the control culture (blue line) and in the presence of TH (1 mM, red line, n = 93). (**d**) Areas under the curves (AUC) of [Ca^2+^]_i_ changes in individual neurons during the first 2 min of glutamate application. Glutamate (10 μM) was applied in Mg^2+^-free buffer containing 10 μM of glycine. The changes in [Ca^2+^]_i_ are presented as the ratio of the fluorescence intensities Ca^2+^ indicator Fura-FF excited at 340 and 380 nm (F340/F380). TH (1 mM) presented in the buffer during whole experiment until addition of protonophore FCCP (1 μM). Significance was analyzed by two-tailed *t*-test, *p < 0.05, **p < 0.01, ***p < 0.001, ****p < 0.0001. (For interpretation of the references to colour in this figure legend, the reader is referred to the Web version of this article.)

To further test whether the action of OGDHC is mediated by NMDA receptors, which are predominantly coupled to Ca^2+^ influx, we determined the effects of glutamate in combination with TH on the changes in intracellular Ca^2+^ levels ([Ca^2+^]_i_) in primary cultures of the rat brain cortex. Individual response of single neurons to 10 μM glutamate was assessed by Fura-2 measurements of cytosolic free Ca^2+^ (Fig. 7a). Feasibility of measurements was verified by adding 1 mM ethylene glycol-bis(β-aminoethyl ester)-N,N,N,N-tetra acetic acid tetrasodium salt (EGTA) for complexing Ca^2+^, and 1 μM *p*-triflouromethoxyphenylhydrazone (FCCP) for uncoupling mitochondria. Maximal intracellular [Ca^2+^] was verified by treatment with ionomycin. In long term experiments, we found that glutamate caused a quick but moderate increase in [Ca^2+^]_i_ (Fig. 7b) followed by a later secondary rise in [Ca^2+^]_i_ (Fig. 7c). The presence of TH substantially decreased the magnitudes of the [Ca^2+^]_i_ in response to glutamate (Fig. 7c). This decrease in [Ca^2+^]_i_ was significant already within first seconds after treatment, when compared to the condition of glutamate alone (Fig. 7d) and remained unchanged for the following 50 min. The total amount of Ca^2+^ released from intracellular stores was not different upon treatment with TH (Fig. 7c).

**Fig. 8:**
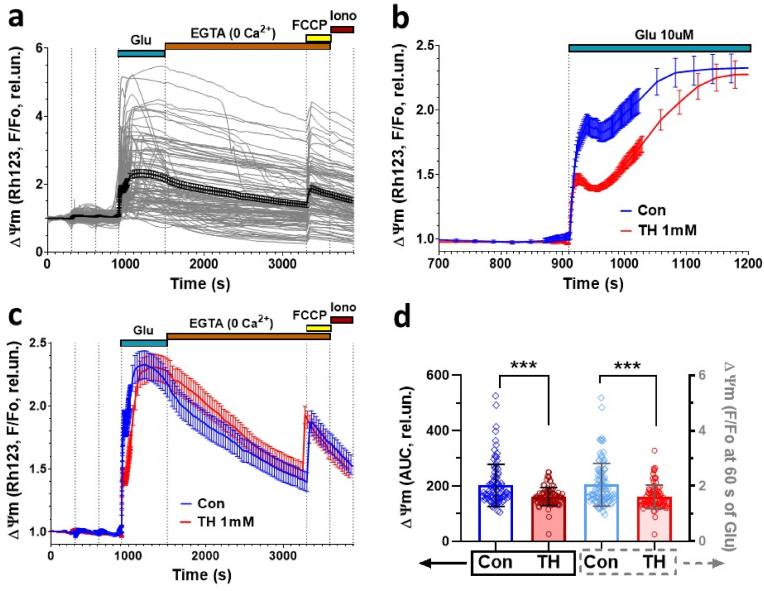
The effect of thiamine (TH) on the glutamate (Glu) induced changes in mitochondrial membrane potential (ΔΨm) in the cultured rat cortical neurons. The ΔΨm changes are presented as the fluorescence intensities ratio (F/Fo) of potential-sensitive probe Rh123 (excitation 500/24, emission 578/105 nm) and normalized to its value at the beginning of the experiment (Fo). (**a**) Changes of mitochondrial potential (ΔΨm) in individual neurons (thin grey curves) and average traces (bold lines) in control culture (glutamate without TH) (n = 101 cells; error bars correspond to SEM). (**b**) X-scale expansion of ΔΨm changes during the first 2 min of glutamate administration (ΔΨm signals were monitored at 0.8 Hz frequency; the rest of experiment the recordings were carried out every 30 s). (**c**) Average changes of ΔΨm (Mean ± SEM) of neurons in response to glutamate (10 μM, glycine 10 μM, Mg^2+^-free) application in the control culture (blue line) and in the presence of TH (1 mM, red line, n = 93). (**d**) Areas under the curves of ΔΨm changes in individual neurons during the first 2 min of glutamate application (AUC, left axis) and amplitudes of F/Fo ratio at 60 s after glutamate addition (right axis). Thiamine (1 mM) presented in the buffer during whole experiment until addition of protonophore FCCP (1 μM). Shown are the results of one of 4 experiments performed with cell cultures prepared on different days (total n > 700). Significance was analyzed by two-tailed *t*-test, ***p < 0.001. (For interpretation of the references to colour in this figure legend, the reader is referred to the Web version of this article.)

Our experiments (Fig. 2) suggested that OGDHC affects glutamate metabolism but not oxidative phosphorylation. To verify this assumption, we determined mitochondrial membrane potential in response to 10 μM glutamate in the same cultured cells (Fig. 8a).

Adding 10 μM glutamate caused immediate decrease in ΔΨm (rapid increase in Rh123 fluorescence) in neurons (Fig. 8b and c), followed by a long-term secondary decrease in ΔΨm, corresponding to secondary increase of [Ca^2+^]_i_ (Fig. 7b), so called delayed calcium deregulation [32,33].

The fluorescence signals of Rh123 during the first 2 min of glutamate administration showed a rather complex pattern, therefore, to estimate the effect of TH on glutamate-induced changes in ΔΨm, we compared the area under curves (AUC) and amplitudes (F/Fo) of Rh123 responses. Both approaches gave similar results (Fig. 8d). TH reduced mitochondrial depolarization in the first 2 min after treatment with glutamate and delayed the secondary decrease in ΔΨm, which is reflected in a more gradual increase in Rh123 fluorescence (Fig. 8b and c), however, TH did not affect maximal mitochondrial depolarization observed in 5 min after glutamate application (Fig. 8b and c).

Removal of glutamate caused a gradual decline in the average Rh123 fluorescence (Fig. 8a,c), which reflects two processes: (1) recovery of ΔΨm, uptake of Rh123 by energized mitochondria resulting in self-quenching of the probe fluorescence, and (2) leak of Rh123 out of the cells due to depolarization of cell plasma membranes and their higher permeability for the probe [34,35].

We did not observe a significant difference in the dynamics of the decrease in Rh123 signals between the control and TH-containing buffers (Fig. 8a, c). Application of the protonophore FCCP (1 μM) at the end of glutamate treatment led to a rapid increase in Rh123 fluorescence, which is associated with the release of the probe from the mitochondria into the cytosol and the unquenching of its fluorescence [34,35].

TH did not significantly affect either the amplitude of the FCCP-induced increase in F/Fo or the AUC during the first 2 min of FCCP application. Probably, reuptake of Rh123 by mitochondria and its leak from the cells during washout of glutamate were approximately equally effective both in the presence and in the absence of TH.

These data show that TH restores glutamate-induced calcium release and mitochondrial depolarization. Thus, TH improves mitochondrial function by enhancing glutamate consumption. Taken together, our data suggest that glutamate toxicity and neuronal death, which results from elevated levels of NO in stressed neurons, can be limited by TH supplementation. It appears therefore that therapeutic TH supplementation might prove effective in any condition of inhibited mitochondrial function via activation of OGDHC and an enhance glutamate consumption.

## 3 Discussion

**Scheme 1:**
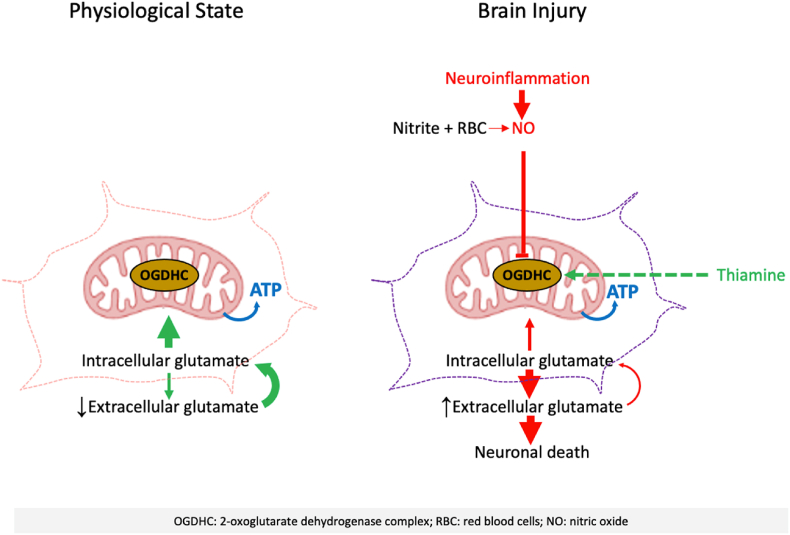
**Working scheme illustrating OGDHC-mediated pathway regulating neuronal death.** Detailed scheme comparing glutamate metabolism in physiological and in injured brain. One pathological state that is activated after an injury is neuroinflammation. Therefore, the increased production of nitric oxide (NO) can target important enzymes of the TCA-cycle in mitochondria (OGDHC) and reduces its activity. In addition, other factors that could be presented in critical brain injury patients, like higher accumulation of nitrite and subarachnoid hemorrhage leads to the production of NO, which could slightly decrease OGDHC activity but not sufficient to completely inhibit the enzyme. The dysfunction of OGDHC indirectly increases the excretion of glutamate into the extracellular space to toxic levels, mediating neuronal cell death. The addition of thiamine, an intracellular precursor of the oxoglutarate dehydrogenase complex (OGDHC) coenzyme, promotes the OGDHC function. In the physiological state, the OGDHC function and the glutamate fluxes are adjusted in such a way, that even higher glutamate concentrations in the intracellular space are not toxic.

The data presented in the current report show that low concentrations of NO occurring in injured brain inhibit OGDHC but do not impair OxPhos. The presence of RBCs, known to bind NO, can slightly improve the activity of OGDHC impaired by hypoxia, while the presence of clinically relevant nitrite concentrations and RBCs reverse this effect, decreasing the average OGDHC activity (Scheme 1). It is possible that the beneficial effect of RBCs is due to binding of endogenously formed NO. Of note, it may well be possible that in patients with SAH, Hb, which is released from RBC, exhibits peroxidase activity that further aggravates damage of neuronal and other cells [36,37] and thus cause a further release of Damage-Associated Molecular Patterns (DAMPs) and glutamate.

The involvement of OGDHC was dissected in neuronal culture using its coenzyme precursor TH and inhibitor SP. TPP synthesized in cytoplasm from TH is the coenzyme of not only OGDHC, but also PDH. In contrast, SP selectivity inhibits OGDHC [25]. Remarkably, actions of SP and TH interact in such a way that OGDHC inhibition may be compensated by increased TH transport to the brain [38]. The inhibition of OGDHC reduces the mitochondrial uptake of glutamate by mitochondria, leading to the accumulation of glutamate in the cytoplasm (Scheme 1). In the cytoplasm of neurons, glutamate is always distributed between energy (taken by mitochondria) and vesicle pools in correspondence with relative affinities of glutamate to the mitochondrial [*K*_m_ = 0.2 mM [39]] and the vesicular (VGLUT) [*K*_m_ = 2 mM [40]] glutamate transporters. Thus, at high levels of cytoplasmic glutamate, its transport into mitochondria will be saturated, in contrast to the vesicular transport which thus acquires the major significance under these conditions. However, the pool of glutamate vesicles in neurons is not homogeneous. There are three pools of glutamate vesicles in neurons, (i) readily releasable pool (RRP), (ii) recycling pool and (iii) reserve pool [41]. These pools have quite different functions serving for evoked and spontaneous release of glutamate. The evoked release is well known and serves neuronal signal transmission [42], while the spontaneous release is less explored, but is associated with glutamate excitotoxicity [43]. It has been shown that evoked glutamate release originates from RRP, while glutamate is spontaneously released from the resting pool [21,44]. Evoked release occurs exclusively in the synapse, while the spontaneous glutamate release occurs in the plasma membrane away from synapses [21] and activates extra-synaptic glutamate receptors, which mediate excitotoxic effects of glutamate.

Several reports support our findings that glutamate uptake by mitochondria is limited by OGDHC activity, which is a critical regulator switching between excitotoxicity and generation of energy from glutamate. Of note, this excitotoxic mechanism occurs independently of the glutamate-mediated neurotransmission, which is regulated by glutamine/glutamate cycle and involves RRP and synaptic receptors. Increased levels of extracellular glutamate block glutamate/cystine transporter leading to the depletion of cysteine and glutathione in the cells. As a result, this can induce ferroptotic cell death as has been previously suggested [22,45]. Thus, inhibition of OGDHC appears to trigger both excitotoxic and ferroptotic neuronal death in injured brain, whereby both are induced by elevated levels of extracellular glutamate.

In conclusion, here we describe a novel mitochondria-mediated pathway of neuronal death, which is based on insufficient activity of the glutamate-dependent segment of the TCA cycle, rather than on impaired OxPhos. This pathway can occur in various neurological diseases accompanied by neuroinflammation and moderately increased tissue levels of NO generated by microglia and other immune cells. Moderately elevated NO inhibits OGDHC (Scheme 1), triggering the redirection of the glutamate flow from the TCA cycle into reserve vesicle pools and their spontaneous release into the extracellular fluid, favoring excitotoxic effects and neuronal death. In addition, our study shows that these effects could be prevented by high doses of TH for treating acute neurological diseases. Currently, the 250 mg/day is a regular dose of TH used in clinical settings, 1.5 g/day is used for successful treatment of patients with Wernicke Korsakoff syndrome [46]. In an animal model of brain injury, beneficial effects of TH were observed at a daily dose of 400 mg/kg in rats [30] corresponding to 64 mg/kg in humans [47]. This dose is comparable to the one used in our current cellular experiments (1 mM thiamine hydrochloride, 337 mg/L). Toxic effects of TH are not known at these doses. The current findings lay the scientific foundation for future clinical trials of high doses of vitamin B1 or its membrane-penetrating pharmacological forms to treat patients suffering from glutamate toxicity due to various forms of brain pathologies.

## 4 Methods

### 4.1 Examination of SAH patients

The study was approved by ethical committee of the Medical University of Vienna (reference number 1871/2014/Amendment). Patients were recruited retrospectively from a prospective collected database approved by the local ethics committee. All patients between 2016 and 2020 of our neurosurgical intensive care unit suffering aneurysmal SAH and with cerebral microdialysis probe implantation prior to day 3 after the bleeding were included.

The microdialysis catheter with a membrane length of 10 mm and a molecular mass cut-off of 20 kDa (70 MD, M Dialysis AB, Stockholm, Sweden) was inserted into the frontal white matter 1–2 cm anterior to Kocher's point into the watershed of the anterior and middle cerebral artery either ipsilateral to the ruptured aneurysm or on the side with maximal extension of subarachnoid blood in case of anterior communicating artery or basilar tip aneurysms. The catheter was perfused at a flow rate of 0.3 μl/min by a precision pump (107 Microdialysis Pump, M Dialysis AB, Stockholm, Sweden) filled with Perfusion Fluid CNS (M Dialysis AB, Stockholm, Sweden). Microdialysis sampling was started not before 3 h after probe implantation to exclude neurochemical changes due to the insertion trauma. Cerebral microdialysate was collected every hour in microvials (MDialysis AB, Stockholm, Sweden) and immediately analyzed bedside (ISCUSflex, M Dialysis AB, Stockholm, Sweden) for glutamate concentrations.

To determine cerebral glutamate levels during early brain injury, glutamate concentrations on day 3 after the bleeding were averaged for each patient. Day 3 after SAH was chosen, as microdialysis data is usually not available before this day in the clinical setting due to delayed probe implantation after the bleeding.

Computed tomography scans were reviewed for new cerebral infarctions (delayed cerebral ischemia) within the first three weeks following SAH. Functional outcome was assessed 3 months after the bleeding using the modified Rankin scale (mRS). A mRS of 0–2 was classified as “good” and a mRS of 3–6 as “poor” functional outcome.

To address this point, we examined twenty patients suffering severe aneurysmal SAH with a median Hunt&Hess grade of 4 (IQR 4–5). The median patients’ age was 53 years (IQR 43–58.25 years) with a male to female ratio of 1:2.3. Microdialysis enabled us to determine glutamate levels in the brain tissue.

### 4.2 Preparation of rat cortex homogenate

The Ludwig Boltzmann Institute for Traumatology complies with the Guide for the Care and Use of Laboratory Animals of the National Institute of Health (revised 2011) and the European directive 2010/63/EU, as well as under Austrian Law TVG 2012. It is authorized by the Municipality of Vienna under the registration number MA58-821/90. This permits extraction of tissues from intact experimental animals. Male Sprague Dawley rats (Charles River, 300–350 g) were anesthetized with 3% isoflurane and sacrificed by decapitation. Cerebral cortex was immediately excised from the brain and dissected into four slices through a vertical and horizontal incision. Blood vessel structures and hippocampus were removed prior to homogenization. 100 mg of cortex tissue were homogenized (RW1 basic homogenizer, IKA, USA) with 3 vol (1:4 wt/vol) of preparation buffer (0.25 M saccharose, 10 mM Tris, 0.5 mM EDTA, 5 mg/ml fatty acid-free bovine serum albumin), pH 7.2. All reagents were obtained from Sigma-Aldrich (USA). These homogenates were used for estimation of mitochondrial respiration, activities of the complexes I and II and NO release.

To assay the intramitochondrial enzymes, the brain homogenates were sonicated in an ultrasonication bath (Thermo Fisher Scientific, CA) for 30 s at maximum power (1.5 kJ); sonication was repeated 3 times with 30 s gaps. The enzymes solubilization was completed by adding one volume of 4-fold concentrated RIPA buffer, that is, 40 mM Tris-HCl buffer, pH 7.4, including 600 mM NaCl, 4 mM EDTA, 1% sodium deoxycholate, and 4% NP-40, to the three volumes of the sonicated homogenate.

### 4.3 Mitochondrial respiration

Mitochondrial activity was measured using a high-resolution respirometer (Oxygraph-2k, Oroboros Instruments, Austria) by incubation of 80 μL homogenate in 2 ml buffer containing 105 mM KCl, 5 mM KH_2_PO_4_, 20 mM Tris-HCl, 0.5 mM EDTA and 5 mg/ml fatty acid-free bovine serum albumin (pH 7.2, 37 °C). State 3 respiration was monitored by addition of substrates for complex I (10 mM glutamate) or complex II (10 mM succinate + complex I inhibitor rotenone (1 ng/ml)), and 1 mM ADP. All reagents were obtained from Sigma-Aldrich (Vienna, Austria). State 3 respiration was inhibited by the addition of NO (saturated solution), and peroxynitrite (Cayman Chemical, USA) at concentrations indicated in the figure legends. NO saturated solution was prepared by bubbling NO gas (AGA Co., Austria) through physiological saline solution (Fresenius Kabi, Austria). NO concentration was determined with a NO analyzer (Sievers 280i, General Electrics, UK).

### 4.4 Determination of NO complexes

EPR samples for NO–Hb and NO–Fe calibration curves were prepared either from RBC or cortex homogenate or their mixture. EPR spectra were recorded at liquid nitrogen temperature (−196 °C) with a Magnettech MiniScope MS 200 EPR spectrometer (Magnettech, Berlin, Germany) in a quartz finger-type Dewar flask filled with liquid nitrogen as previously described [48].

### 4.5 Experiments on B35 neuroblastoma cells

B35 rat neuroblastoma cells were obtained from the American Type Culture Collection (ATCC, VA). B35 cells were maintained in Dulbecco's Modified Eagle Medium (DMEM, Invitrogen, CA) containing 4.5 g/L^−1^ glucose supplemented with 10% fetal bovine serum (FBS, Hyclone, UT), 4 mM glutamine, 100 IU·mL^−1^ penicillin and 100 μg mL^−1^ streptomycin (Invitrogen, CA) at 37 °C in 5% CO_2_ atmosphere in collagen-coated tissue culture vessels (BioCoat™ Collagen I culture flasks, Becton Dickinson BioSciences/Falcon, MA). Cells were cultured on 24-well plates coated with 0.01% poly-d-Lysine (PDL, Sigma-Aldrich, USA), 0.5 × 10^5^ cells/cm^2^. Cells were kept in growth media to adhere overnight (37 °C in 5% CO_2_), and the following day were induced by combination of serum deprivation and chemical induction afforded by change in media containing 1% (w/v) bovine serum albumin and 10 μM all-trans retinoic acid (ATRA) instead of 10% FBS. Cell culture medium was changed on day 3 to a modified differentiation medium that replaced 1% bovine serum albumin with 1% FBS to minimize cell loss during differentiation while maintaining conditions of relative serum starvation. On the fifth day of differentiation, cells were subjected to *in vitro* injury.

### 4.6 LDH release assay

Lactate dehydrogenase (LDH) release was measured by mixing cell culture supernatant (30 μL) with 100 μL LDH assay reagent containing 110 mM lactic acid, 1350 μM nicotinamide adenine dinucleotide (NAD+), 290 μM N-methylphenazonium methyl sulphate (PMS), 685 μM 2-(4-iodophenyl)-3-(4-nitrophenyl)-5-phenyl-2H-tetrazolium chloride (INT) and 200 mM Tris (pH 8.2). The kinetics of changes in absorbance (492 nm) were recorded for 15 min (kinetic LDH assay). LDH activity values were determined as maximal velocity of NADH formation (mOD/min).

### 4.7 Immunocytochemistry in murine primary mesencephalic culture

Pregnant OF1/SPF mice (Institute for Laboratory Zoology and Veterinary Genetics – Himberg, Austria) were housed and handled in accordance to the legislation for the protection of animals used for scientific purposes (Directive 2010/63/EU).

### 4.8 Preparation of murine primary dissociated mesencephalic cultures

On gestation day 14, embryos were taken under aseptic conditions for the preparation of mesencephalic primary cultures. Therefore, each following step was executed in a new Petri dish containing fresh Dulbecco's phosphate buffered saline (DPBS; Invitrogen, UK). Embryonic brains were cut out and the midbrains excised. Thereafter, the meninges were removed cautiously and the brain tissue was dissected and trypsinised (0.1% in DPBS; Invitrogen, UK). Furthermore, the cells were treated with DNase (0.02% in HBSS buffer; Roche, Germany) for 7 min at 37 °C. Afterwards, the cell pellet was overlaid with 3 ml basic medium (DMEM supplemented with Hepes buffer (10 mM; Sigma-Aldrich, Germany), d-glucose (30 mM; Sigma-Aldrich, Germany), glutamine (4 mM; Sigma-Aldrich, Germany), penicillin–streptomycin (10 U/ml and 0.1 mg/ml, respectively; Roche, Germany) and heat-inactivated FCS 10% (Sigma-Aldrich, Germany)), gradually triturated using fire-polished Pasteur pipettes. After a sedimentation time of 10 min, the supernatant was transferred into an Erlenmeyer flask containing 6 ml of basic medium and the cell pellet resuspended in 3 ml basic medium. The trituration and sedimentation step were repeated two more times, resulting in 15 ml cell suspension which was diluted with basic medium to a final concentration of 7.5 × 10^5^ cells/ml and seeded onto poly-d-lysine (50 μg/ml in DPBS; Sigma-Aldrich, Germany) pre-coated 48-well plates (340 μl per well). Dissociated cells were incubated at 37 °C in a humidified atmosphere containing 5% CO_2_. The medium was exchanged on the first and the third days *in vitro*. Half of the medium was replaced with serum-free DMEM containing 0.02 ml B-27/ml DMEM on the fifth day and again the medium was changed with B27 (Invitrogen, Germany)/DMEM medium on day 6. After 24 h, cultures were treated for 15 min with glutamate alone (0.01, 0.1, 0.5 and 1 mM; Sigma-Aldrich, Germany) or pretreated with TH (1 mM; Calbiochem, Germany) or memantine (60 μM; Sigma-Aldrich, Austria) for 1 h followed by a co-treatment with glutamate (0.1, 0.5 and 1 mM) for 15 min. After two more days of cultivation, anti-tyrosine-hydroxylase staining was performed.

### 4.9 Anti-tyrosine-hydroxylase immunocytochemistry

Cells were stained using the Vectastain ABC peroxidase kit according to the manufacturer's instructions with slight modifications (Vector Laboratories, Burlingane, CA, USA). In brief, cells were fixed with 4% paraformaldehyde fixative (Sigma-Aldrich, Darmstadt, Germany) solution for 15 min at 4 °C. Cell permeabilization was done with Triton-X (0.4% in DPBS) for 30 min at room temperature (RT). Between the following steps, cells were washed with DPBS. Horse serum (5% in DPBS) was used to block unspecific binding sites. The plates were allowed to incubate for 90 min at RT. Following this, the anti-tyrosine-hydroxylase antibody (1:1000 diluted in horse serum solution) was administered at 4 °C overnight, and the secondary biotinylated antibody (horse anti-mouse IgG; 1:200 in DPBS) for 90 min at RT. Meanwhile, a 1 + 1 mixture of avidin and biotin solution was prepared and allowed to stand for a minimum of 30 min at RT to ensure sufficient AB-complex formation. Afterwards, the AB-complex solution was mixed 1:500 with DBPS and applied for another 90 min at RT. The reaction product was developed in a solution of diaminobenzidine (1.4 mM) in PBS containing hydrogen peroxide (3.3 mM). Cells were mounted with Kaiser's glycerol gelatine and tyrosine hydroxylase immunoreactive cells were counted with a Nikon inverted microscope with a 10 × objective (Nikon Instruments Europe BV, Netherlands). After anti-TH staining, neurite lengths were measured. For this reason, photographs of six cells per condition and well were taken under an inverted microscope. The pictures were edited with Adobe Photoshop CS5.1 and the neurite lengths calculated by pixel evaluation. A calibration standard was used for reference.

### 4.10 Dopaminergic cell count and neurite outgrowth measurements

Cells were mounted with Kaiser's glycerol gelatine (Merck, Germany) and tyrosine hydroxylase immunoreactive cells were counted with a Nikon inverted microscope with a 10 × objective (Nikon Instruments Europe BV, Netherlands). After anti-TH staining neurite lengths were measured. For this reason, photographs of six cells per condition and well were taken under an inverted microscope. The pictures were edited with Adobe Photoshop CS5.1 and the neurite lengths calculated by pixel evaluation. A calibration standard was used for reference.

### 4.11 Experiments on culture of NG108-15 cell line

*Mus musculus* neuroblastoma and *Rattus norvegicus* glioma hybrid cell line NG108-15 (ATCC, VA) was cultured at 37 °C and 5% CO_2_ in DMEM with 4.5 g/L glucose (Lonza Bioscience, Switzerland) supplemented with 10% FBS (Sigma-Aldrich, Germany) and 100 IU/ml^−1^ penicillin and 100 μg/ml^−1^ streptomycin (Gibco, CA). Passaging was performed at the confluence of 80% following the standard cell culturing technique, with the exception of cell detachment being carried out mechanically instead of enzymatically, due to the low adherence rate of the cells. Cell numbers were determined by manual counting on a Neubauer hemocytometer (Gizmo Supply, CA); trypan blue dye (Gibco, CA) was used for viability staining.

OGDHC inhibition and activation treatment was introduced to confluent cells (seeding density 0.5 × 10^5^ cells/cm^2^), after allowing a 24 h period for attachment. During the attachment and treatment period, supplementation with l-glutamine was omitted in order to prevent endogenous production of glutamate from glutamine. Cells were treated with 5 mM glutamate (Sigma-Aldrich, Germany), 1 mM TH (Sigma-Aldrich, Germany), 0.5 mM NO-donor DETA-NONOate (Cayman Chemical, MI) and 0.2 mM SP (MedChem Express, NJ) over 48 h. Cell pellets were collected for determination of viability, OGDHC and PDH activity; culture supernatants were collected for detection of extracellular glutamate concentration and LDH release.

### 4.12 Determination of OGDHC and GDH activities in cells

OGDHC activity was determined by monitoring reduction of NAD^+^ as part of the reaction of 2-oxoglutarate turnover catalyzed by the enzyme. Firstly, cell pellets were resuspended in homogenization buffer (0.05% BSA, 0.5 mM EDTA, 1% protease inhibitor cocktail, 250 mM sucrose, 10 mM TRIS) in order to reach a total volume of the buffer and cells of 300 μL, and were then lysed in an ultrasonication bath (Thermo Fisher Scientific, CA) for 30 s at maximum power; sonication was repeated 3 times with 30 s gaps. Lysates were incubated on ice with RIPA buffer for 20 min after 4-times concentrated RIPA is diluted 4-fold by a homogenate. Reaction mix consisted of 200 μL incubation buffer (1 mM calcium chloride, 0.05 mM coenzyme A, 1 mM dithiothreitol (DTT), 1 mM magnesium chloride hydrate, 50 mM MOPS, 2.5 mM NAD^+^; pH adjusted to 7.2), 50 μL cell lysate, 2.8 mM 2-oxoglutarate and 0.62 mM TPP. All reagents were obtained from Sigma-Aldrich, Germany. Measurement was conducted on a black 96-well culture plate (Sigma-Aldrich, Germany), kinetically monitoring fluorescence of the reaction product NADH at 460 nm, with the excitation wavelength of 340 nm. The activity of glutamate dehydrogenase (GDH) was measured spectrophotometrically at 340 nm by monitoring the decrease of the NADH production in the reaction mixture containing 100 mM TRIS/HCl pH 7.5, 2.5 mM 2-oxoglutarate, 0.2 mM NADH and 50 mM NH_4_Cl. A calibration was performed measuring the fluorescence of a dilution series of NADH.

### 4.13 Determination of extracellular glutamate

To determine glutamate levels in culture supernatants and fresh treatment media, a method based on detection of the reduced form of 3-acetylpyridine adenine dinucleotide (APAD^+^), an analog of NAD^+^, as a product of glutamate oxidation by GDH was employed. 200 μL reaction mix containing 80 μL sample, 0.8 mM APAD+, 45 U/mL GDH, 135 mM potassium chloride and 74 mM TRIS (all reagents obtained from Sigma-Aldrich, Germany) were incubated on a heating block (Eppendorf, Germany) with orbital shaking at 37 °C and 800 rotations per minute for 2 h. Measurement was conducted on a clear 96-well culture plate (Corning, NY) at 37 °C, kinetically detecting the absorbance peak of APADH at 366 nm. A serial dilution of glutamate was used to obtain a calibration curve. Glutamate concentration in the sample was calculated using the slope of the absorbance increase in the sample over time expressed in minutes multiplied by the slope of the calibration curve.

### 4.14 Determination of PDH activity

Pyruvate dehydrogenase complex (PDH) activity was determined by detecting NADH formation from NAD^+^ catalyzed by the enzyme; additionally, LDH was inhibited in order to prevent its interference by NADH consumption. Inhibition was achieved by a catalytic site-targeting agent GNE-140. Sample preparation was performed as described in the methods for detection of OGDHC activity. Incubation buffer contained 1 g/L BSA, 0.1 mM coenzyme A, 0.3 mM DTT, 5 mM l-carnitine, 1 mM magnesium chloride, 2.5 mM NAD^+^, 50 mM potassium phosphate and 0.2 mM TPP; pH was adjusted to 7.5. GNE-140 stock was prepared in the concentration of 10 mM in DMSO (final concentration 0.09 mM); pyruvate stock was prepared in the concentration of 250 mM, with the final concentration being 11.5 mM. All reagents were obtained from Sigma-Aldrich, Germany. 200 μL incubation buffer, 2 μL GNE-140 stock and 5 μL sample were incubated at room temperature on a black 96-well plate (Sigma-Aldrich, Germany) for 20 min; 10 μL pyruvate stock were added directly before the measurement. NADH fluorescence was kinetically detected at 460 nm with the excitation wavelength of 340 nm; PDH activity was derived from the relative increase in fluorescence signal of the sample over time expressed in minutes, multiplied by the slope of the calibration curve obtained from the fluorescence values of a dilution series of NADH.

### 4.15 Primary culture of rat cortical neurons for the detection of Ca^2+^

Experiments with animals were performed in accordance with the ethical principles and regulatory documents recommended by the European Convention on the Protection of Vertebrate Animals used for experiments (Guide for the Animals and Eighth Edition, 2010), as well as in accordance with the “Good Laboratory Rules practice”, approved by order of the Ministry of Health of the Russian Federation No. 199n of January 04, 2016. All the protocols were approved by the Ethics Committees at the Institute of Theoretical and Experimental Biophysics, Russian Academy of Sciences (Protocol No. December 2020 of February 17, 2020).

Primary cultures of rat brain cortical neurons were prepared from the cortex of one-day old Wistar rats. The rats were anesthetized, decapitated, and the cortex was removed and separated from the meninges. The extracted tissues were washed with a Ca^2+^- and Mg^2+^-free Hanks solution, dissected, incubated in a papain solution (15 min, 36 °C) and dissociated in fresh minimum essential medium (MEM). The homogeneous suspension was centrifuged two times (200 g, 5 min). The pellet was resuspended to a concentration 10^6^ cells/mL in the neurobasal medium (NBM) supplemented with B-27 Supplement and penicillin/streptomycin. Aliquots of the cell suspension (200 μl) were transferred onto coverslips attached to the wells of 35-mm glass bottom Petri dishes (MatTeck, Ashland, MA), or, alternatively, 400 μl aliquots were transferred into wells of a 24-well plastic plate. The cells were kept in an incubator at 37 °C, 95% air +5% CO_2_, and a relative humidity of 100%. Cytosine arabinoside (5 μM) was added to the medium in 2–3 days to prevent proliferation of glial cells and to obtain cultures with a percentage of neurons ≥90%. The cells were supplied with nutrients every three days by replacing 1/3 of the medium with a fresh one. The cultures were used in experiments 10–12 days after plating.

### 4.16 Measuring [Ca^2+^]_i_ and ΔΨm in the cultures of cortical neurons

To measure changes of the intracellular free Ca^2+^ concentration ([Ca^2+^]_i_), cortical neurons were loaded with a low-affinity Ca^2+^ indicator Fura-FF in the form of acetoxymethyl ester (Fura-FF/AM, 4 μM, 60 min at 37 °C). A non-ionic detergent PluronicF-127 (0.02%; Molecular Probes, United States) was added to facilitate the Fura-FF/AM penetration into the cells. Fura-FF fluorescence was excited alternately at 340/26 and 387/11 nm and recorded at 578/105 nm (dichroic mirror 514 nm). For simultaneous measurements of [Ca^2+^]_i_ and ΔΨm, cells were also loaded with Rhodamine 123 (Rh123; 2.5 μg/ml, 15 min at 37 °C); Rh123 fluorescence was excited at 500/24 and recorded and 578/105 nm. Accumulation of Rh123 in energized mitochondria is accompanied by self-quenching of the dye; depolarization of the organelles results in release and dequenching of Rh123 (Duchen, 2003)). The measurements were performed at 25–29 °C in a medium containing (mM): 135 NaCl, 5 KCl, 2 CaCl_2_, 1 MgCl_2_, 20 HEPES, 5 d-glucose; pH 7.4. Glutamate (100 μM) was applied in a Mg^2+^-free buffer containing 10 μM of glycine. In Ca^2+^ -free buffers, CaCl_2_ was replaced with 0.1 mM EGTA and 2 mM MgCl_2_. To assess the glutamate-induced accumulation of Ca^2+^ in mitochondria, it was released from organelles by depolarization with a protonophore carbonyl-p- (trifluoromethoxy) phenylhydrazone (FCCP, 1 μM). The maximal response of Fura-FF was calibrated with the Ca^2+^ ionophore ionomycin (Iono, 2 μM in the presence of 5 mM CaCl_2_). Fluorescence measurements were performed using a fluorescence imaging system, which included a Nikon Ti2 Eclipse inverted microscope (Japan) equipped with a 20x/NA = 0.45 fluorite objective, a pE-340fura LED illumination system (CoolLED, UK), а set of light filters, and a Prime BSI Express sCMOS Camera (Teledyne Photometrics, USA) operated by Nikon NIS-Elements software (Japan). The fluorescence of Fura-FF and Rh123 in neurons was recorded in the cell soma (in the cytoplasm and nucleus) and was corrected for the background fluorescence, which was measured in the areas of the culture devoid of axons and dendrites. The recordings were performed with a frequency of 1 frame/30 s, with the exception of 30 s before the application of glutamate and during the first 120 s after its introduction; for these 150 s images were recorded at a rate of 1 frame/1.2 s to better comply with electrophysiological measurement protocols. Fura-FF/AM and Rh123 were purchased from ThermoFisher (USA).

## Author contributions

H·B., V.E.K, V.B. and A.V.K. conceived and designed the study. A.H. collected and analyzed the clinical data. All experiments were performed by A.W. and N.M. with the following exceptions: L.R. performed mitochondrial respiration analyses; J.C.D., L.T. and R.M. performed experiments on the morphology of neurons; G.T. and C.S. performed experiments with B35 cells; A.V.G. performed experiments on NO release; G.M., V.B. and L.T. established methods on the TCA enzymatic activity; R.R.S., A.M.S.,I.A.K. and V.G.P. prepared primary culture of rat cortical neurons and performed experiments on [Ca2+]i and mitochondrial potential changes. A.W., N.M., V.E.K. and A.V.K. wrote the manuscript with input from all authors.

## Declaration of competing interest

The authors declare that they have no known competing financial interests or personal relationships that could have appeared to influence the work reported in this paper.

## Data Availability

Data will be made available on request.
